# Knockdown of CLIC4 enhances ATP-induced HN4 cell apoptosis through mitochondrial and endoplasmic reticulum pathways

**DOI:** 10.1186/s13578-016-0070-1

**Published:** 2016-01-25

**Authors:** Haowei Xue, Jinsen Lu, Renxiang Yuan, Jinli Liu, Yehai Liu, Kaile Wu, Jing Wu, Juan Du, Bing Shen

**Affiliations:** Department of Oral and Maxillofacial Surgery, The First Affiliated Hospital of Anhui Medical University, Hefei, 230022 Anhui China; Department of Physiology, Anhui Medical University, 81 Meishan Road, Hefei, 230032 Anhui China; Department of Dermatology, Anhui Provincial Hospital, Hefei, 230001 Anhui China; Department of Otorhinolaryngology, Head and Neck Surgery, The First Affiliated Hospital of Anhui Medical University, Hefei, 230022 Anhui China

**Keywords:** Chloride intracellular channel 4, Head and neck squamous carcinoma, Apoptosis, Mitochondrial membrane potential, Endoplasmic reticulum stress, Adenosine triphosphate

## Abstract

**Background:**

Human head and neck squamous carcinoma is the 6th most prevalent carcinoma worldwide. Although many novel therapies have been developed, the clinical treatment for patients remains non-ideal. Chloride intracellular channel 4 (CLIC4), one of the seven members of the CLIC family, is a newly found Cl^−^ channel that participates in various biological processes, including cellular apoptosis and differentiation. Accumulating evidence has revealed the significant role of CLIC4 in regulating the apoptosis of different cancer cells. Here, we investigated the functional role of CLIC4 in the apoptosis of HN4 cells, a human head and neck squamous carcinoma cell line.

**Results:**

In the present study, we used immunohistochemical staining to demonstrate that the expression level of CLIC4 is elevated in the tissue of human oral squamous carcinoma compared with healthy human gingival tissue. Specific CLIC4 small interfering RNA was used to knockdown the expression of CLIC4. The results showed that knockdown of CLIC4 with or without 100 μM adenosine triphosphate (ATP) treatment significantly increased the expression of Bax, active caspase 3, active caspase 4 and CHOP but suppressed Bcl-2 expression in HN4 cells. Moreover, the results from the TdT-mediated dUTP nick end labeling assay indicated that CLIC4 knockdown induced a higher apoptotic rate in HN4 cells under the induction of ATP. In addition, knockdown of CLIC4 dramatically enhanced ATP-induced mitochondrial membrane depolarization in HN4 cells. Moreover, intracellular Ca^2+^ measurement revealed that Ca^2+^ release induced by ATP and thapsigargin, a Ca^2+^-ATPase inhibitor of the endoplasmic reticulum, was significantly enhanced by the suppression of CLIC4 in HN4 cells.

**Conclusions:**

Knockdown of CLIC4 enhanced ATP-induced apoptosis in HN4 cells. Both the pathways of mitochondria and endoplasmic reticulum stress were involved in CLIC4-mediated cell apoptosis. Based on our finding, CLIC4 may be a potential and valuable target for the clinical treatment of head and neck squamous carcinoma.

**Electronic supplementary material:**

The online version of this article (doi:10.1186/s13578-016-0070-1) contains supplementary material, which is available to authorized users.

## Background

Head and neck cancer, the 6th most common carcinoma worldwide, severely deteriorates the health of elderly people [1]. Despite recent advances in therapy, clinical treatment for patients with head and neck squamous cell carcinoma (HNSCC) remains to be improved further [2]. Thus, exploring new ways to improve the treatment for HNSCC is required and of great significance [3]. In recent years, accumulating evidence has indicated the role of ion channels in the development of different cancers [4, 5]. Among various ion channels, the Cl^−^ intracellular channel (CLIC) has been listed as one of the most active ones [5, 6]. CLIC is the most recently discovered family of Cl^−^ channels. CLIC4, also known as p64H1, RS43, or mtCLIC, is one of the seven members of the CLIC family and is ubiquitously expressed in the brain, liver and skin at a particularly high level [5, 7, 8]. The literature has shown that the subcellular localization of CLIC4 varies among different cell types, including the inner mitochondrial membrane, cytoplasm, Golgi apparatus and endoplasmic reticulum (ER) [8].

Recent studies have shown that CLIC4 participates in the cell apoptotic process. However, the results are quite complex with a controversial effect [8]. Both suppression and overexpression of CLIC4 can induce apoptosis in keratinocytes [8]. Additionally, the apoptotic response mediated by CLIC4 occurs not only through the mitochondrial pathway accompanied with the increase of Bax/Bcl-2, activation of caspase 3 and release of cytochrome C, but through the ER stress pathway along with the increase in CHOP and cleaved caspase 4 expression [9]. Multiple studies have investigated the expression profile and regulatory effect on the apoptosis of CLIC4 in cancer cells [8, 10–13]. It has been suggested that the suppression of CLIC4 via CLIC4-antisense inhibits the growth of human osteosarcoma cells in vitro and in vivo, weakens cell proliferation and increases cell apoptosis [14]. However, other studies have reported that the loss of CLIC4 is a common phenotype in many human cancers and marks malignant progression; additionally, if CLIC4 was introduced into human breast cells, tumor growth is inhibited [15]. Therefore, the expression profile and regulatory effect on apoptosis of CLIC4 in each specific cancer cell may be different. Moreover, the effect of CLIC4 on HNSCC apoptosis has not yet been examined. Adenosine triphosphate (ATP) released by stressed and dying cells [16] is a potent extracellular apoptosis inducer that disrupts normal cellular metabolism by binding extracellular specific receptor and regulating intracellular metabolic products [17]. Because CLIC4 is indicated as an integral part of the cellular stress-response pathway and is a significant regulator of cell apoptosis and growth in live cells, we hypothesize that the regulation of CLIC4 protein expression may affect ATP-induced apoptosis.

The present study was undertaken to investigate the expression profile of CLIC4 in the surgical specimens of HNSCC patients and the effect of CLIC4 knockdown on ATP-induced apoptosis in the HNSCC cell line HN4.

## Results

### Enhanced CLIC4 expression in HNSCC tissue

**Fig. 1:**
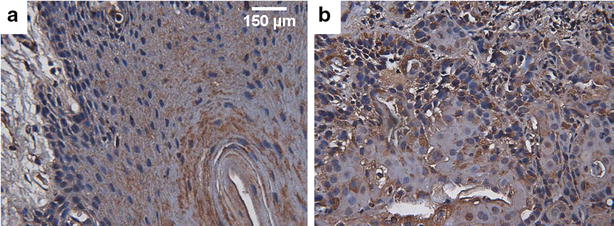
Expression levels of CLIC4 in human normal gingival and oral squamous carcinoma. Immunohistochemical staining images showing the expression level of CLIC4 in human normal gingival tissue (**a**) and human oral squamous carcinoma (**b**). CLIC4 protein was recognized by anti-CLIC4 mouse monoclonal primary antibody

Because growing evidence has indicated that CLIC4 is involved in the development of different cancers, including cutaneous cancer, breast cancer, ovarian cancer and others [11, 12, 15], we also investigated CLIC4 expression in surgical specimens from HNSCC patients. Immunohistochemical staining showed that CLIC4 expression in HNSCC tissue was obviously higher than that in normal gingival tissue (Fig. 1). Based on this finding, we hypothesized that CLIC4 might also have a relationship with the development of HNSCC.

### Knockdown of CLIC4 enhances ATP-induced HN4 cell apoptosis via the mitochondrial pathway

**Fig. 2:**
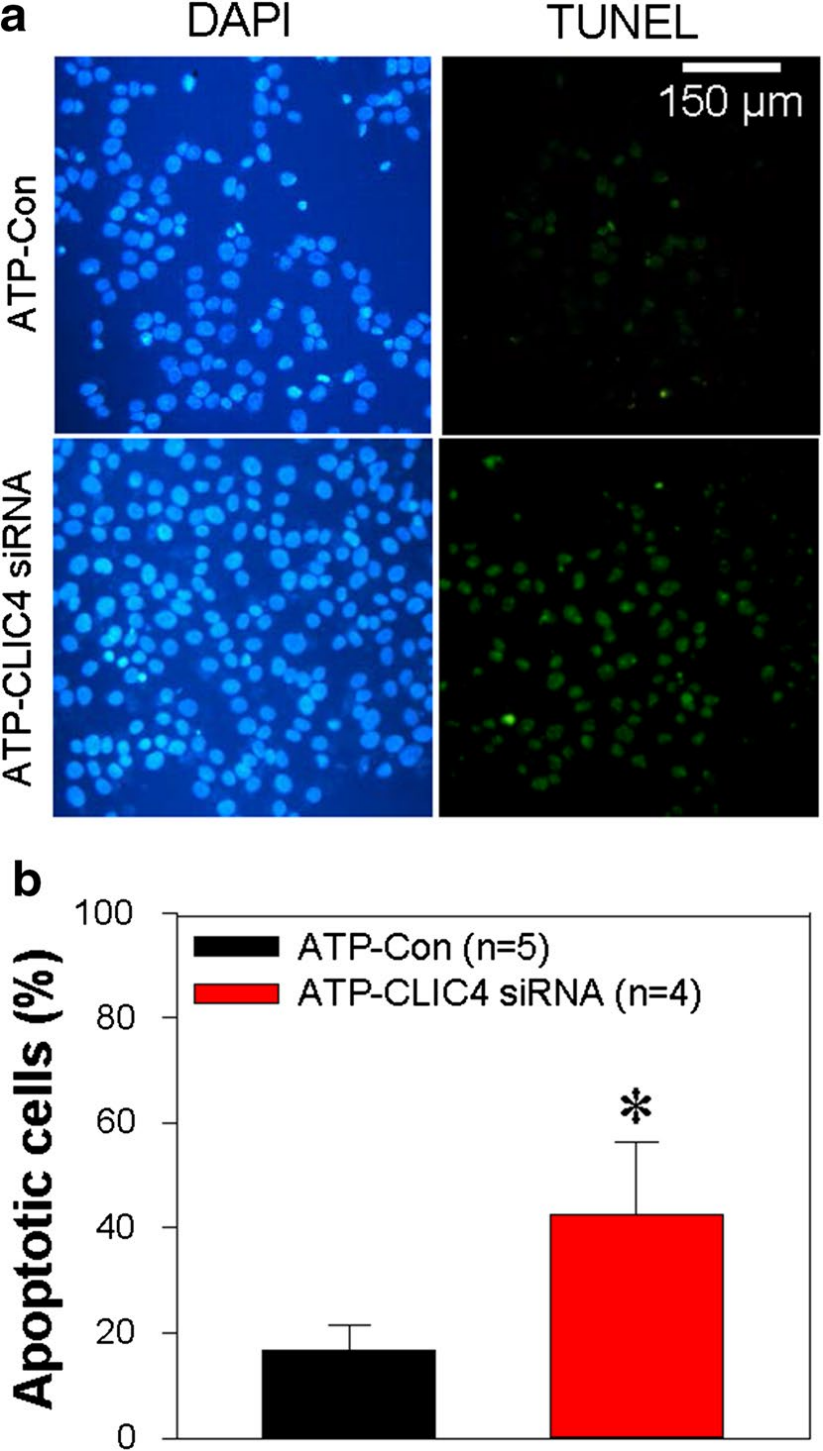
Effect of CLIC4 on the apoptosis of HN4 cells. **a** Representative images showing the cellular nucleus (DAPI) and apoptotic cells (TUNEL). The HN4 cells were transfected with CLIC4 (ATP-CLIC4 siRNA) or scrambled (ATP-Con) siRNA and treated with 100 μmol/L ATP for 3 h to facilitate apoptosis. **b** Summarized data showing the percentage rate of the apoptotic cells. The percentage of the apoptotic cells = apoptotic cell number/total cell number. Values are shown as the mean ± SE. n = 4–5. *P < 0.05 vs. the control (ATP-Con) group

**Fig. 3:**
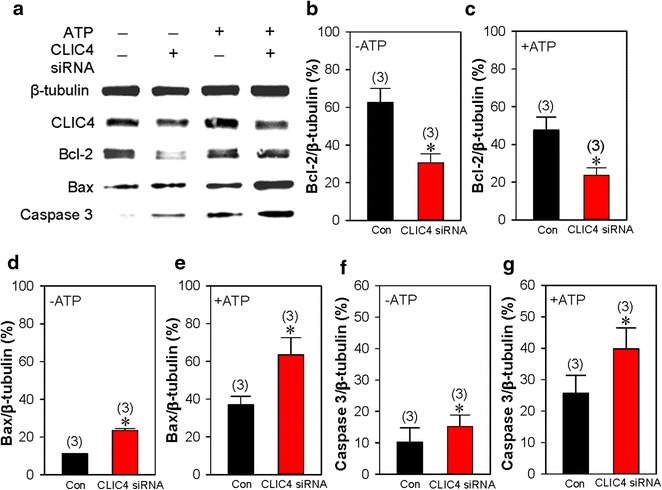
Effect of CLIC4 on the mitochondrial apoptotic pathway. Representative images (**a**) and summarized data (**b–g**) showing the expression levels of CLIC4 (**a**), Bcl-2 (**a**, **b**–**c**), Bax (**a**, **d**–**e**) and active caspase 3 (**a**, **f**–**g**). HN4 cells were transfected with CLIC4 (+, CLIC4 siRNA) or scrambled (−, Con) siRNA, and treated with (+, +ATP) or without (−, −ATP) 100 μmol/L ATP for 3 h. β-Tubulin was used as a loading control. Values are shown as the mean ± SE. n = 3. *P < 0.05. vs. the control (Con) group

Recent studies have reported that CLIC4 was a regulator for cancer cell apoptosis [8]. Therefore, we employed specific small interfering RNA (siRNA) to suppress CLIC4 expression to identify the role of CLIC4 in HN4 cell apoptosis. Our data indicated that CLIC4 siRNA significantly suppressed CLIC4 expression (Figs. 3a, 6a and Additional file 1: Figure S1). TdT-mediated dUTP nick end labeling (TUNEL) assay was utilized to detect apoptotic cells, and 100 μmol/L ATP was used to evoke cell apoptosis. Our data showed that the percentage of apoptotic cells was significantly increased in CLIC4-specific siRNA-transfected cells compared with scrambled siRNA-transfected ones (Fig. 2a, b).

It is well documented that mitochondrial signaling pathway crucially participates in cell apoptosis [18]. To elucidate the intracellular signaling pathway, we first examined the expression change in apoptosis-related proteins within the mitochondrial pathway. Immunoblotting data suggested that with or without 100 μmol/L ATP treatment, knockdown of CLIC4 via specific siRNA notably increased the expression of Bax and active caspase 3, which are the proverbial pro-apoptotic proteins, but decreased the expression of Bcl-2, which demonstrates as an anti-apoptotic effect, compared with scrambled siRNA (Fig. 3).

**Fig. 4:**
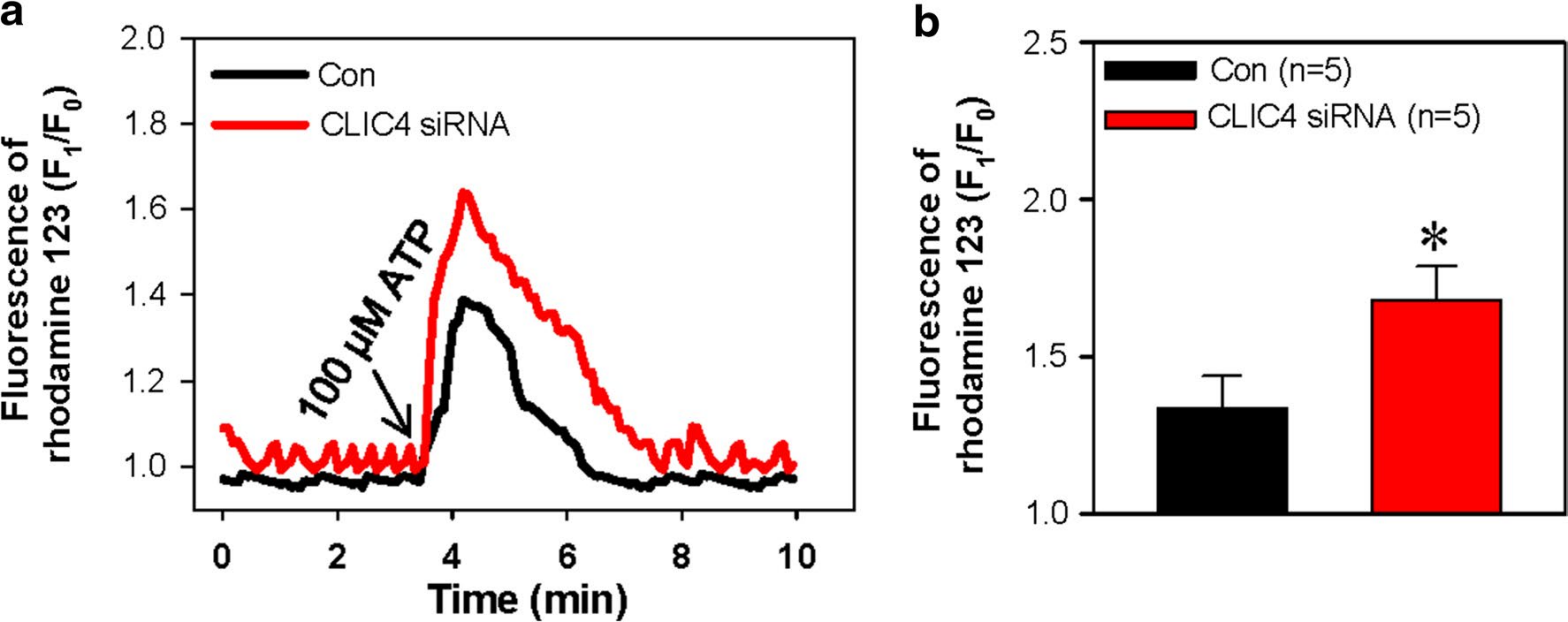
Effect of CLIC4 on ATP-induced mitochondrial membrane depolarization. Traces (**a**) and summarized data (**b**) showing 100 μmol/L ATP-induced changes in the HN4 cell mitochondrial membrane potential, which was detected by the cell-permeable membrane potential sensitive-fluorescent indicator rhodamine-123. The change in the membrane potential was indicated by the ratio of the fluorescence density before and after the application of ATP. The ratio increase represents the membrane potential depolarization. The HN4 cells were transfected with CLIC4 (CLIC4 siRNA) or scrambled (Con) siRNA. Values are shown as the mean ± SE. n = 5. *P < 0.05. vs. control (Con) group

The reduction of the mitochondrial membrane potential or enhanced mitochondrial membrane depolarization is an early sign of cell apoptosis ahead of DNA breakage and activation of caspase 3 [19]. ATP can increase [Ca^2+^]_i_ and induce mitochondrial membrane depolarization [20]. Therefore, we assessed whether knockdown of CLIC4 affected ATP-induced mitochondrial membrane depolarization. The mitochondrial membrane potential was detected by rhodamine-123, which is a membrane potential-sensitive fluorescent indicator. Our results showed that the application of 100 μmol/L ATP induced mitochondrial membrane depolarization. Compared with scrambled siRNA, CLIC4 siRNA transfection dramatically enhanced ATP-induced mitochondrial membrane depolarization (Fig. 4a, b). These findings strongly indicate that knockdown of CLIC4 enhances ATP-induced HN4 cell apoptosis through the mitochondrial apoptotic pathway.

### Knockdown of CLIC4 enhanced ATP- and TG-induced intracellular Ca^2+^ release and ER stress-mediated apoptosis

**Fig. 5:**
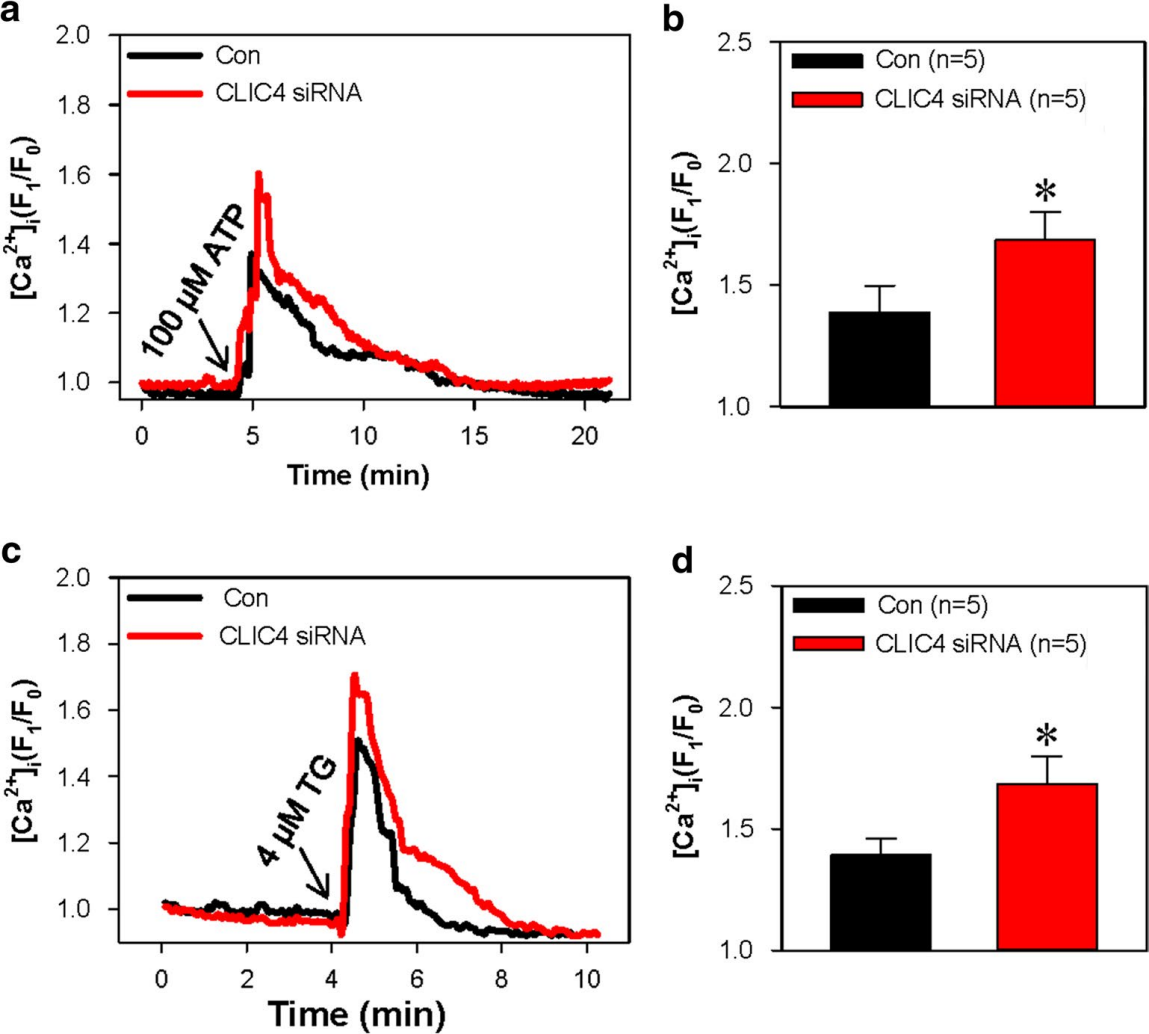
Effect of CLIC4 on the Ca^2+^ store release. Representative traces (**a** and **c**) and summarized data (**b** and **d**) showing 100 μmol/L ATP− (**a** and **b**) and 2 μmol/L thapsigargin (TG, **c** and **d**) -induced HN4 cell intracellular Ca^2+^ ([Ca^2+^]_i_) rise in Ca^2+^-free saline solution. [Ca^2+^]_i_ was detected by the cell-permeable Ca^2+^-sensitive fluorescent indicator Fluo-8 AM. The change in [Ca^2+^]_i_ was indicated by the ratio of the fluorescence density before and after the application of agonists. The HN4 cells were transfected with CLIC4 (CLIC4 siRNA) or scrambled (Con) siRNA. Values are shown as the mean ± SE. n = 5. *P < 0.05. vs. the control (Con) group

**Fig. 6:**
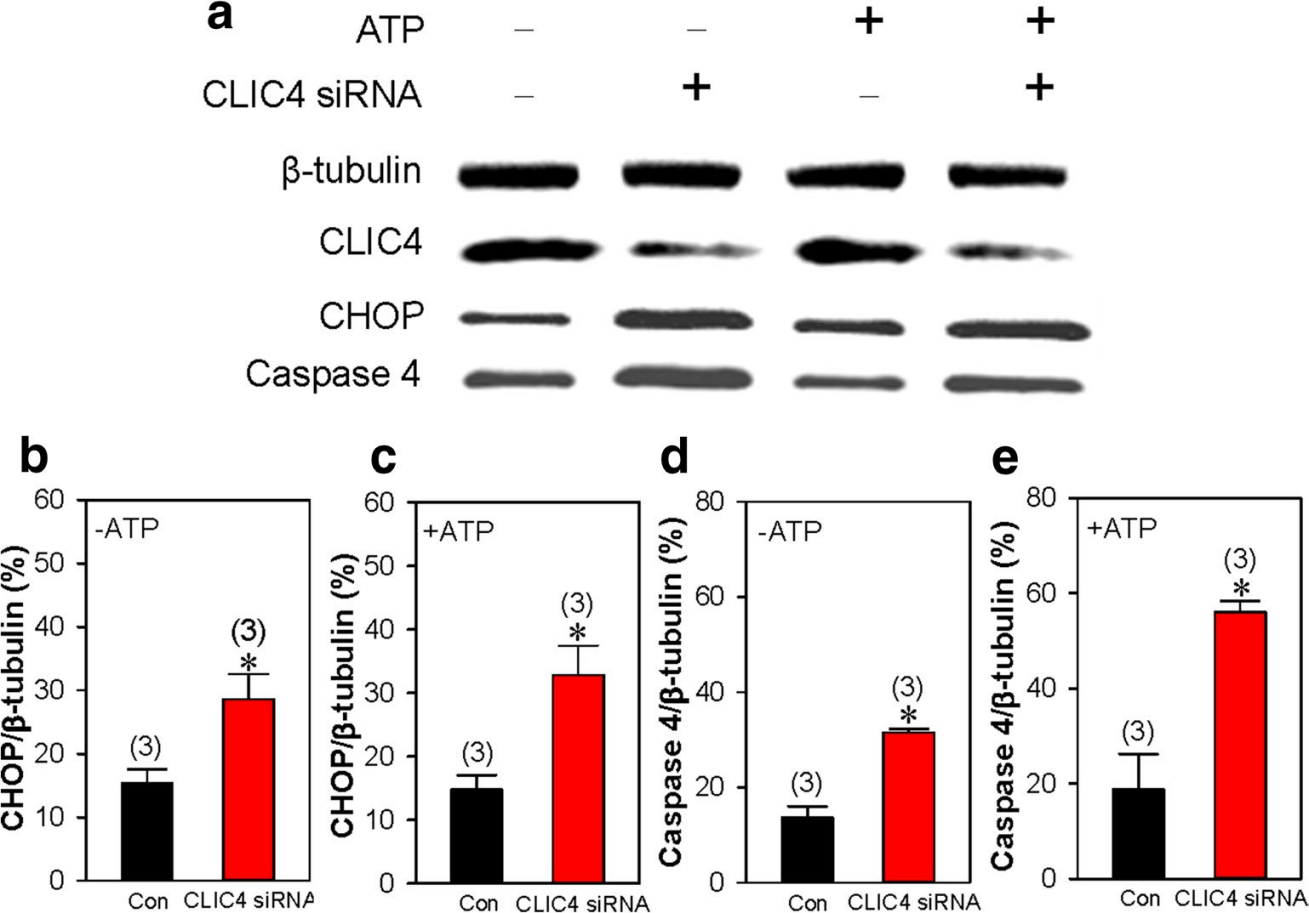
Effect of CLIC4 on the endoplasmic reticulum-stress apoptotic pathway. Representative images (**a**) and summarized data (**b**–**e**) showing the expression levels of CLIC4 (**a**), CHOP (**a**, **b**–**c**) and active caspase 4 (**a**, **d**–**e**). The HN4 cells were transfected with CLIC4 (+,CLIC4 siRNA) or scrambled (−, Con) siRNA, and treated with (+, +ATP) or without (−, −ATP) 100 μmol/L ATP for 3 h. β-Tubulin was used as a loading control. Values are shown as the mean ± SE. n = 3. *P < 0.05. vs. the control (Con) group

Besides being distributed in the mitochondrial membrane, CLIC4 is also located on the membrane of ER, which is an alternative mediator of apoptosis. Previous studies have suggested that the depletion of ER Ca^2+^ stores leads to cell apoptosis and growth arrest [21, 22]. The Ca^2+^ stores can be depleted by both ATP and thapsigargin (TG), an ER Ca^2+^-ATPase inhibitor [20, 23]. Therefore, we examined the effect of CLIC4 on the ATP- and TG-induced Ca^2+^ release from ER Ca^2+^ stores. Our results showed that knockdown of CLIC4 significantly enhanced both ATP- and TG-induced Ca^2+^ release in HN4 cells, indicating that Ca^2+^ stores might be overloaded by the loss of CLIC4 (Fig. 5a–d). Because accumulating evidence has revealed that heavy and continued ER stress may activate the process of cell apoptosis [24], we hypothesize that ER stress may also participate in the apoptosis induced by CLIC4 knockdown. To address this issue, in HN4 cells transfected with CLIC4 or scrambled siRNAs and with or without ATP treatment, we examined the expression level of CHOP and active caspase 4, both of which play key roles in ER stress-induced apoptosis [9]. Data showed that knockdown of CLIC4 markedly elevated the expression level of CHOP and active caspase 4 in HN4 cells compared with the scrambled siRNA group regardless of the presence of ATP (Fig. 6). Together, these results indicate that ER stress along with CHOP and caspase 4 activation also participates in ATP-induced apoptosis of HN4 cells following CLIC4 knockdown.

## Discussion

In the present study, we investigated the functional role of CLIC4, a member of the CLIC family, in the apoptosis of HN4 cells. The major findings in our study are as follows: (1) the CLIC4 expression level was elevated in HNSCC tissue compared with normal gingival tissue; (2) knockdown of CLIC4 via specific siRNA significantly enhanced ATP-induced apoptosis of HN4 cells; (3) knockdown of CLIC4 dramatically increased the expression level of Bax, CHOP, active caspase 3 and active caspase 4, but decreased Bcl-2 expression with or without ATP treatment; (4) suppression of CLIC4 notably strengthened ATP-induced mitochondrial membrane depolarization; (5) Ca^2+^ measurement data indicated that knockdown of CLIC4 significantly enhanced ATP- and TG-induced Ca^2+^ release. Taken together, our findings demonstrated that CLIC4 negatively regulated HN4 cell apoptosis via the mitochondrial and ER stress pathways.

HNSCC is one of the six most prevalent cancers that threatens the health of the global population with approximately 600,000 new cases annually [1–3]. Conventional treatment no longer satisfies a better cure rate; therefore, seeking new therapeutic approaches is of great need and significance [2, 25]. It is well documented that Cl^−^ channels undertake essential tasks to maintain the hemostasis of the internal environment and play important roles in biological processes, including the regulation of the cell membrane potential, acidification of organelles, cell proliferation and apoptosis [6]. Among various Cl^−^ channels, CLIC, a newly found class of Cl^−^ channels, was suggested to participate in cancer development and apoptosis [5]. More importantly, CLIC4 gained more attention for its participation in malignant transformation, cellular stress and apoptosis of cancer cells [5]. Several studies have suggested that CLIC4 regulates cancer cell apoptosis and proliferation even it displays an opposite effect in different cancer cells [7–10, 12, 14, 15]. Our immunohistochemical staining and TUNEL assay showed that CLIC4 was elevated in HNSCC tissue, and knockdown of CLIC4 via the transfection of specific siRNA enhanced ATP-induced apoptosis in HN4 cells. ATP is able to be released from the stressed and dying cells [16], for example the cells in the central part of solid tumor. In cancer cells, chemotherapy also can elicit ATP release [26]. Therefore, the results indicated that the loss of CLIC4 may inhibit the development of HNSCC and strengthened curative effect of chemotherapy.

CLIC4 is widely distributed inside cells, particularly on the membrane of two essential organelles, mitochondria and ER, both of which are involved in two major apoptotic pathways [5, 8, 9]. In the mitochondrial pathway, Bcl-2 family proteins undertake an essential task during the regulation of apoptosis. The fate of cells is determined by the ratio of pro-apoptotic and anti-apoptotic Bcl-2 family members [27]. Activated Bax forms oligomers and inserts into the mitochondrial membrane causing dissipation of the mitochondrial membrane potential and cytochrome C release [28]. Subsequently, the apoptotic cascade is activated, whereas Bcl-2 prevents Bax activity via exerting its pro-apoptotic effect [28]. A previous study revealed that the suppression of CLIC4 enhanced the apoptosis induced by H_2_O_2_ in glioma C6 cells along with the dissipation of the mitochondrial membrane potential and nuclear translocation of CLIC4 but without the change of Bax/Bcl-2 ratio [10]. In our study, we found that CLIC4 knockdown not only dramatically elevated the expression level of Bax and active caspase 3 but notably down-regulated the Bcl-2 expression with or without ATP treatment in HN4 cells, revealing that Bax/Bcl-2 ratio participated in CLIC4-mediated apoptosis. The mitochondrial membrane potential, which is formed by the normal function of proton pumps plays key roles in maintaining oxidative phosphorylation and ATP production [29]. Enhanced mitochondrial membrane depolarization is a well-recognized sign of apoptosis in the early stage [20]. Our data show that the knockdown of CLIC4 significantly enhanced ATP-induced mitochondrial membrane depolarization in HN4 cells, further proving that the knockdown of CLIC4 exerts a pro-apoptotic effect through the mitochondrial apoptosis pathway and strengthened ATP-induced apoptosis in HN4 cells.

A recent study reported that the inhibition of CLIC4 triggers mitochondria- and ER stress-mediated apoptosis in human glioma U251 cells under starvation [9]. ER stress, which refers to a subcellular pathological state of the ER manifesting as Ca^2+^ dyshomeostasis and the accumulation of unfolded proteins, is essential in the process of cell apoptosis [30–32]. Our [Ca^2+^]_i_ measurement results suggested that the knockdown of CLIC4 enhanced ATP- and TG-induced intracellular Ca^2+^ release, indicating that Ca^2+^ overload possibly caused by CLIC4 knockdown may contribute to the apoptotic progress of HN4 cells. Heavy and continued ER stress may activate downstream apoptotic signaling molecules, including CHOP and active caspase 4, further leading to cell apoptosis [33–36]. In the present study, we found that the expression levels of ER stress-related proteins, including CHOP and active caspase 4, were strongly increased following the knockdown of CLIC4 with or without ATP treatment. Based on our total findings, the apoptotic response mediated by CLIC4 occurs through both the mitochondrial pathway accompanied with the enhancement of mitochondrial membrane depolarization and activation of caspases 3, and the ER stress pathway with the elevated expression of CHOP and active caspase 4. Accumulating evidence has revealed the synergistic roles played by both the mitochondria and ER in the process of CLIC4-mediated cancer cell apoptosis [9], findings that were consistent with ours.

## Conclusions

In conclusion, we demonstrate that the expression level of CLIC4 was elevated in oral squamous carcinoma tissues, and the knockdown of CLIC4 enhanced ATP-induced apoptosis via both the mitochondrial and ER stress pathways in HN4 cells. Our finding indicates that CLIC4 may be considered as a potential target for treating HNSCC and sheds light on future clinical therapy. But the effect of CLIC4 overexpression in the apoptosis of HN4 cells still remains. The future study will benefit this issue.

## Methods

### Materials

Specific siRNA for human CLIC4 (accession numbers, NM_013943; corresponding to the cDNA sequence 5-GCTGAAGGAGGAGGACAAAGA-3) and scrambled siRNA (5-ACGCGUAACGCGGGAAUUU-3) were designed and obtained from Biomics Company [9]. For cell transfection, Lipofectamine 2000 reagent and opti-MEM I reduced serum medium were purchased from Invitrogen Company. Cell counting kit CCK8 was obtained from Beyotime Company. Anti-β-tubulin, anti-CHOP (GADD-153), anti-Bax, anti-Bcl-2, anti-caspase 3 and anti-caspase 4 rabbit polyclonal primary antibodies and anti-CLIC4 mouse monoclonal primary antibody were purchased from Santa Cruz Biotechnology. The TUNEL bright green apoptosis detection kit was purchased from Vazyme Biotechnology. Rhodamine-123 was obtained from invitrogen.

### Immunohistochemistry

Each specimen was obtained with written informed consent from each patient involved. The procedures were conducted in line with the Declaration of Helsinki and the Good Clinical Practice [37, 38]. The surgical specimens of human normal gingival and oral squamous carcinoma were fixed in 4 % poly-formaldehyde for 1 to 2 d. Next, the specimens were dehydrated and embedded in paraffin following routine methods. Paraffin-embedded tissues were sliced into sections with 5 μm thickness. The specimens were treated by deparaffinization and rehydration routinely. Heat-mediated antigen retrieval was performed with citrate buffer (0.01 M, pH 6.0) in a microwave oven. Specimens were treated for 10 min with 3 % peroxide-methanol for quenching endogenous peroxidase ablation and blocked with 10 % normal goat serum for 20 min at room temperature. Next, the specimens were incubated with mouse anti-CLIC4 primary antibody (1:50, sc-135739, Santa Cruz) overnight at 4 °C. After the specimens were washed out and incubated with goat anti-mouse IgG secondary antibody conjugated with horseradish peroxidase for 30 min, avidin–biotin-peroxidase reagents were added. Peroxidase activity was visualized by incubating the specimens with 3, 3′-diaminobenzidine tetrahydrochloride (DAB, Japan) for 10 min, and hematoxylin was used to visualize the cell nucleus. The negative control was performed by incubating with normal mouse serum to replace the primary antibody. Cells exhibiting brown particle staining were considered positive.

### Cell culture and transfection

HN4, an HNSCC cell line, was derived from patients with HNSCC [39]. HN4 cells were cultured in Dulbecco’s modified Eagle medium (DMEM, 4.5 g/L glucose) (Life Technologies, USA) supplemented with 10 % fetal bovine serum and antibiotics (100 KU/L penicillin and 100 mg/L streptomycin) in an incubator at 37 °C with 5 % CO_2_. HN4 cells were transiently transfected with CLIC4 siRNA using Lipofectamine 2000 following the manufacturer’s instruction and then were cultured for another 48 h before the following experiment.

### CCK8 assay

The viability of the HN4 cells was determined by the CCK8 assay. After transfection with CLIC4 and scrambled siRNA for 1 d, respectively, HN4 cells were trypsinized and seeded in 96-well plates at an equal density of 6 × 10^3^ cells/well. On the following day, after the treatment was based on experimental design, 10 μl of CCK8 was added to each well with 4 h of incubation in an incubator at 37 °C with 5 % CO_2_. The absorbance was recorded at a wavelength of 450 nm. The cell viability is proportional to the value of the absorbance value.

### Immunoblotting

Western blotting was performed as previously described [40]. Briefly, HN4 cells were lysed by lysis buffer containing 20 mM Tris–HCl (pH 7.5), 150 mM NaCl, 1 mM Na_2_EDTA, 1 mM EGTA, 1 % NP-40, 1 % sodium deoxycholate, and 2.5 mM sodium pyrophosphate, plus protease inhibitor cocktail tablets, and total proteins were extracted. Next, 30 μg of proteins was loaded in each well and separated by 12 % SDS-PAGE with a voltage of 100 V for 1 h and then transferred to PVDF membranes. After blocking with 5 % non-fat milk diluted by PBST for 1 h at room temperature, the membrane was incubated at 4 °C overnight with the appropriate primary antibodies. On the following day, the primary antibodies were washed out, and the membrane was incubated with horseradish peroxidase-conjugated secondary antibodies. Each band for specific protein was visualized by ECL. The optical density of each blot was normalized to that of β-actin, analyzed within the same lane and represented as the relative optical density.

### TUNEL assay

Cell apoptosis was detected by the TUNEL assay. Experiments were performed according to the manufacturer’s protocol. Briefly, after appropriate experimental treatment, HN4 cells on the cover slip were fixed with 4 % paraformaldehyde solution and then incubated with proteinase K solution (2 mg/ml) for 5 min at 37 °C to inactivate endogenous peroxidase. Next, the cells were treated with equilibration buffer for 20 min at room temperature. After washing with PBS for three times, the cells were incubated in TdT buffer for another 1 h at 37 °C. Following washing with PBS, HN4 cells were stained with 4′,6-diamidino-2-phenylindole (DAPI) for 10 min. Finally, the cells were dried and analyzed with a fluorescence microscope. Each cell was visualized by blue fluorescence (DAPI) at the wavelength of 460 nm, and the apoptotic cells were identified by green fluorescence at the wavelength of 520 nm. Apoptotic percentage = apoptotic cell number/total cell number × 100 %.

### Mitochondrial membrane potential measurement

Rhodamine-123 was utilized to measure the mitochondrial membrane potential in HN4 cells [41]. HN4 cells transfected with scrambled or CLIC4 siRNAs were trypsinized and seeded onto circular cover glass placed in 6-well plates. On the following day, the cells were incubated with 2 μmol/L rhodamine-123 for 40 min at 37 °C. After washing, the fluorescence intensity of rhodamine-123 was recorded by confocal laser microscopy. The fluorescent dye was exited at 507 nm, and the signal was collected at an emission wavelength of 529 nm. Each data of the fluorescence intensity were the average of 20–30 cells.

### [Ca^2+^] measurement

Intracellular Ca^2+^ concentration ([Ca^2+^]_i_) was measured using methods described elsewhere [42]. Briefly, HN4 cells were seeded on circular coverslips and incubated with 10 μmol/L Fluo-8 with 0.02 % pluronic acid F-127 at 37 °C for 30 min. Ca^2+^ release was evoked by treating HN4 cells with 4 μmol/L TG or 100 μmol/L ATP for 10 min in Ca^2+^-free PBS that contained (in mmol/L) 140 mmol/L NaCl, 1 mmol/L MgCl_2_, 5 mmol/L KCl, 10 mmol/L glucose, 0.2 mmol/L EGTA, and 5 mmol/L HEPES, pH 7.4. The real-time change of fluorescence indicating [Ca^2+^]_i_ was recorded by Nikon fluorescence microscopy with a 488 nm excitation and 515 nm long pass emission wavelength. Changes in [Ca^2+^]_i_ were displayed as the ratio of fluorescence relative to the intensity before the application of ATP or TG (F1/F0). Each data of the fluorescence intensity were the average of 20–30 cells.

### Statistical analysis

Collected data are presented as mean ± SE. The two-tailed independent *t* test was used to compare the results in different groups. A *P* value <0.05 was considered a significant difference. All of the statistical analyses were performed by the software SigmaPlot 11.0.

## Additional file

10.1186/s13578-016-0070-1 Effect of CLIC4 or scrambled siRNA on CLIC4 expression. Effect of CLIC4 or scrambled siRNA on CLIC4 expression. Summarized data showing the expression level of CLIC4. HN4 cells were transfected with CLIC4 or scrambled siRNA, and then were treated with (ATP-Con, ATP-CLIC4) or without (Con, Con-CLIC4) 100 μmol/L ATP for 3 h. β-Tubulin was used as a loading control. Values are shown as the mean ± SE. n = 3. **P* < 0.05. vs. the control (Con) group,^#^ *P* < 0.05 vs. the ATP control (ATP-Con) group.

## Abbreviations

HNSCC: head and neck squamous cell carcinoma
CLIC: Cl^−^ intracellular channel
ER: endoplasmic reticulum
[Ca^2+^]_i_: intracellular Ca^2+^ concentration
siRNA: small interfering RNA
TG: thapsigargin
ATP: adenosine triphosphate
DAPI: 4′,6-diamidino-2-phenylindole
TUNEL: TdT-mediated dUTP nick end labeling
