# Supplementary material for: Knockdown of CLIC4 enhances ATP-induced HN4 cell apoptosis through mitochondrial and endoplasmic reticulum pathways
Source: Cell Biosci. 2016 Jan 25;6:5. doi: 10.1186/s13578-016-0070-1 (PMC4727302; doi:10.1186/s13578-016-0070-1)
Supplement: Supplementary file 1 — 10.1186/s13578-016-0070-1 Effect of CLIC4 or scrambled siRNA on CLIC4 expression. Effect of CLIC4 or scrambled siRNA on CLIC4 expression. Summarized data showing the expression level of CLIC4. HN4 cells were transfected with CLIC4 or scrambled siRNA, and then were treated with (ATP-Con, ATP-CLIC4) or without (Con, Con-CLIC4) 100 μmol/L ATP for 3 h. β-Tubulin was used as a loading control. Values are shown as the mean ± SE. n = 3. *P < 0.05. vs. the control (Con) group,# P < 0.05 vs. the ATP control (ATP-Con) group. [file 13578_2016_70_MOESM1_ESM.pdf]

Knockdown of CLIC4 enhances ATP-induced HN4 cell apoptosis through mitochondrial and endoplasmic reticulum pathways

Haowei Xue[1,#], Jinsen Lu[2,#], Renxiang Yuan[2], Jinli Liu[3], Yehai Liu[4], Kaile Wu[4], Jing Wu[4], Juan Du[2], Bing Shen[2,*]

Additional file 1: Fig. S1

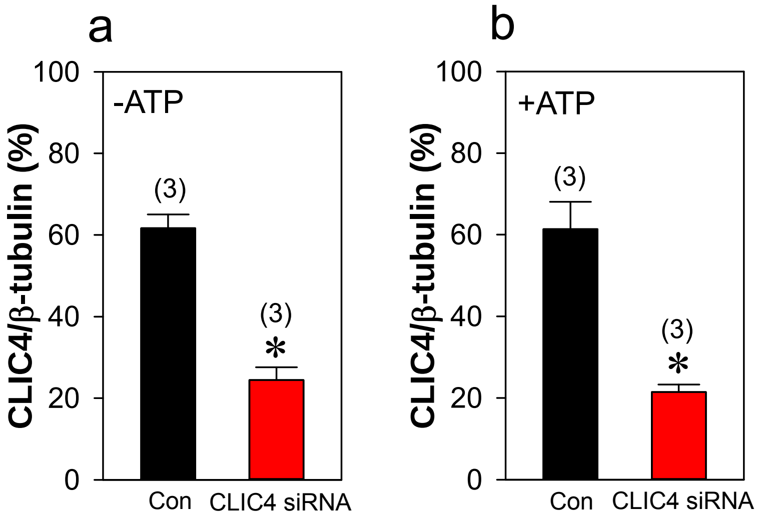

Fig. S1 Effect of CLIC4 or scrambled siRNA on CLIC4 expression. Summarized data showing the expression level of CLIC4. The HN4 cells were transfected with CLIC4 (CLIC4 siRNA) or scrambled (Con) siRNA, and treated without (**a**) or with (**b**) 100 μmol/L ATP for 3 h. β-Tubulin was used as a loading control. Values are shown as the mean ± SE. n = 3. *$P < 0.05$. vs. the control (Con).
